# Modulation of Porcine Gut Microbiota and Microbiome: Hologenomic, Dietary, and Endogenous Factors

**DOI:** 10.3390/pathogens13030225

**Published:** 2024-03-05

**Authors:** Ming Z. Fan, Sung Woo Kim

**Affiliations:** 1 Department of Animal Biosciences, One Health Institute, University of Guelph, Guelph, ON N1G 2W1, Canada; 2 Department of Animal Science, College of Agriculture and Life Sciences, North Carolina State University, Raleigh, NC 27695, USA; sungwoo_kim@ncsu.edu

Global pig production contributes to about 35% of the world’s meat production and consumption [1]. Honeyman [2] comprehensively reviewed the major sustainability issues, including economic, environmental, and social sustainability, that swine production faced in the US. Unfortunately, almost 30 years after, these sustainability issues still persistently exist in the pig industry in the US [3,4] and other advanced industrial countries [5,6] and have been further expanded and intensified in the rest of the global intensive pig production regions [7,8]. On the one hand, this serves as a testimonial that resolving some of the fundamental animal biological questions and associated technological innovations associated with swine production is very challenging; on the other hand, it also calls for swine researchers to have resilience in pursuing their research efforts and for society to consistently give the much-needed and long-term support and investment for research resources. Under this context, the porcine gut microbiota and microbiome have been recognized as the central focus for improvements in pig nutrition, physiology, and thus productivity and health [8,9]. Thus, papers in this Special Issue of *Pathogens* have been collected to reflect and shed light on some of the ongoing active research by the global research community in this topic area.

Fowler et al. [10] investigated the fecal microbiome origins of the large variability in final body weight (BW) of the later finishing swine under a typical US commercial swine production and research facility setting. Their pigs had the same genetic background, were fed the same commercial swine diets, and did not differ between the barrows and the gilts in growth rates and the final market BW [10]. However, while the pen’s mean final BW averaged 133 kg, the heavy final BW group (146 kg) finishers were heavier than the light final BW group (120 kg) by 22% [10]. This large variation in final market BW within the same finisher herd not only limits the swine production profit margin but also reduces feed and nutrient utilization efficiency with negative impacts on the environment. This is because the major negative environmental effects are derived from later liquid swine manure storage and fermentation after feeding; thus, the efficiency of feed and nutrient utilization is proportionally related to the negative impacts on the environment in swine production [11]. Clearly, viewing and approaching the host–microbiome ecosystem as a holobiont at the hologenomic level would help decipher this complexity [12,13,14,15]. Fowler et al. [10] approached this applied swine production issue in the large variability in final BW of the finisher swine via metagenomics. They subsequently carried out a combined total of 757,687 high-quality sequence reads from the V1–V3 partial region of the 16S rRNA genes generated from all the fecal samples of the light (final BW range of 113–129 kg, n = 21) and heavy (final BW weight range of 137–156 kg; n = 23) pigs [10]. Alpha-diversity endpoints were assessed by richness (Chao1, ACE) and diversity (Shannon, Simpson) that were calculated via the obtained operational taxonomic units (OTUs) [10]. Comparisons of the fecal bacterial communities between the light and the heavy barrows were also conducted using principal coordinate analysis (PCoA) of these OTU data [10]. The relative abundance responses of fecal bacterial taxonomy at the phylum and family levels were also compared using the OTUs based on the partial V1–V3 region sequencing of the 16S rRNA genes [10]. However, they were only able to detect significantly higher abundances of two bacterial species of *Clostridium jeddahitimonense* and *C. beijerinckii* that were involved in starch and prebiotic sugar utilization evident from the literature in the light final BW finisher group [10]. It is well established that dietary starch and sugar digestive utilization are not limiting factors in feeder pigs [8]. It is conceivable that the V1–V3 partial region of the 16S rRNA gene sequencing platform that was used in the study by Fowler et al. [10] had a limited resolution to allow for quantifying bacterial relative abundance changes at the species level. The V4 region 16S rRNA gene sequencing and analyses identified a number of bacterial groups at the genus level that were associated with growth efficiency and carcass traits in several pig breeds [16]. Several studies using metagenomic analyses identified a number of specific bacterial species at the cecal and fecal levels that were associated with growth and feed conversion efficiency in pigs [17,18,19,20]. A further expanded catalog of microbial genes and their associated metagenomes, based upon the metagenomic analyses, were also established in the pigs, and this bacterial gene level of data would potentially allow the identification of the gut-specific bacterial genes that account for growth rate, feed conversion efficiency, and carcass quality traits in pigs [21]. Moving forward, q-PCR, several recently emerged high-resolution full-length 16S rRNA gene sequencing platforms, and high-throughput metagenomic sequencing and analyses will enable swine researchers to identify specific gut and fecal microbial species and their genes responsible for the variability in final BW of the finisher swine herd and to develop mitigation strategies for closing this knowledge gap.

The dietary and oral route of enterotoxigenic *Escherichia coli* infection is one of the common opportunistic pathogenic bacterial gut infections in food production animals including pigs and in humans. In this Special Issue, Duarte and Kim [22] orally inoculated the young pigs with F18^+^ *E. coli* in a saline solution on d 7 and observed the piglets’ responses on d 28 post weaning. As anticipated, F18^+^ *E. coli* challenge significantly induced gut dysbiosis, reduced the fecal score, and decreased growth performances (they did not report these actual data) [22]. They used a close-to-full-length V1–V9-region 16S rRNA gene sequencing platform and observed differential bacterial alpha- and beta-diversity responses as well as bacterial taxonomy abundance changes at the family, genus, and species levels in the jejunal digesta and in the fecal samples, respectively [22]. They further showed that F18^+^ *E. coli* infection caused jejunal mucosal upper villus atrophy with a significantly reduced villus height and villus-to-crypt-depth ratio as a revealed biological mechanism in the weanling pigs subjected to F18^+^ *E. coli* infection [22]. The potential use of a high-resolution full-length 16S rRNA gene sequencing platform will reveal more relative abundance changes in bacterial taxonomy at the species level in the jejunal digesta and at the fecal level in weanling piglets with F18^+^ *E. coli* infection.

Guar gum, along with beta-glucan and pectin, are classified as viscous soluble fibers and are regarded as anti-nutritive factors, because, at significant dietary levels, these fibers can reduce growth performances by decreasing nutrient utilization efficiency, food passage rate, and food intake [23,24,25]. On the other hand, these viscous soluble fibers have been exploited as potential functional food ingredients; for example, dietary guar gum at 10–20% was shown to induce hypoglycemia [23], hypocholesterolemia [23,26,27], and enhanced anti-inflammatory cytokine IL-10 abundance in the colon [28], thus having health management implications in humans and companion animals. In this Special Issue, Inoue et al. [29] took a different approach and investigated the non-nutritive gut modifier prebiotic aspect of the enzymatically hydrolyzed galactomannan polysaccharide guar gum at 0.06% (Wt/Wt) from the weanling to the finisher phase in pigs. While the authors did not report chain length and viscosity changes of the enzymatically hydrolyzed guar gum, they did confirm the efficacy of dietary supplementation of the partially hydrolyzed guar gum at 0.06% in inducing increased in vivo fecal organic acid concentrations [29]. The authors did not report significant differences in fresh carcass weight at the 76 kg live market weight; however, dietary supplementation of the guar gum at 0.06% reduced the total number of days needed in reaching their market BW [29]. Furthermore, the authors did not report any growth performance endpoints such as feed intake, average daily gain, and feed conversion efficiency, while they did observe differential responses in the fecal bacterial alpha-diversity and relative abundance responses of taxonomy at the phylum and genus levels using the V3 region 16S rRNA gene sequencing platform. While the data from the study by Inoue et al. [29] provided evidence of the prebiotic effect potential of the hydrolyzed guar gum at 0.06%, further research is needed to examine the needed growth performance and the relative abundance responses of fecal bacterial taxonomy at the bacterial species level.

In this Special Issue, Scott et al. [30] reviewed the literature evidence of dietary original and dietary supplementation of exogenous polyphenolic compounds as antimicrobial, anti-oxidative, and anti-inflammatory bioactive compounds with implications in both animal and human nutrition and the health space. And this is consistent with literature research reports and reviews in this topic area [31,32,33]. As potential therapeutic veterinary biologics, the effects, efficacy, and working mechanisms of various sources and doses of polyphenolics need to be defined and established under a relevant porcine veterinary pathogenic and clinical setting in following the concerned governmental regulatory registration and approval guidelines. As a potential non-nutritive gut modifier feed additive, the effects, efficacy, and working mechanisms of various sources and doses of polyphenols need to be defined and established in pigs, for example, in weanling pigs, under a close-to-commercial swine production condition, and in following the concerned governmental regulatory registration and approval guidelines, e.g., the Canadian Food Inspection Agency Regulation Guidance-1 Regulatory Guidance of Feed Registration Procedures and Labelling Standards [34]. Once the governmental regulatory review and approval of a polyphenolic-based non-nutritive gut modifier feed additive have been granted by a governmental regulatory agency in one country, for example, by the CFIA in Canada, further commercial registration, labeling, and commercial applications for the polyphenolic-based non-nutritive gut modifier feed additive in Canada and other countries would be relatively straight forward. Furthermore, responses to antimicrobial resistance (AMR), AMR gene abundances, and the relative abundances of fecal bacterial taxonomy at the bacterial species levels will likely be anticipated for such a governmental regulatory registration and approval process for the clarification of related impacts on the environment.

As a group of the major endogenous digestive enzymes, gut alkaline phosphatases (APs), including the intestinal AP isoform (IAP) and the tissue non-specific AP isoform (TNAP), are expressed on the gut apical membrane along the porcine small-large intestinal tract [35]. Gut APs contribute to the dephosphorylation of the lipid moiety of endotoxin and other pathogen-associated-molecular pattern (PAMP) member molecules such as the endotoxin lipopolysaccharides (LPSs) and triphosphate nucleotides, in promoting a balanced gut commensal microbiota and microbiome, thus maintaining gut eubiosis and preventing inflammation and metabolic endotoxemia [35]. Previous studies showed that weaning-associated growth retardation and gut mucosal villus atrophy were associated with decreased gut AP digestive capacity and affinity in the pig [36]. In this Special Issue, Yin et al. [35] evidently reviewed that gut AP affinity is more limiting and is the bottleneck in the dephosphorylation of gut luminal PAMP compounds. They further showed that *N*-deglycosylation of IAP and TNAP along the porcine small–large intestinal longitudinal axis could effectively modulate the plasticity of weaned porcine gut AP functionality in terms of the maximal activity and affinity of these AP enzymes. Optimal gut microbiota and microbiome improve not only the efficiency of the digestive utilization but also the post-digestive utilization of dietary energy and nutrients [8,37,38,39]. The effects of dietary supplementation of the hydrolyzed guar gum at 0.06% on the gut microbiome responses [29]; the F18^+^ *E. coli*-challenge-induced gut dysbiosis and differential jejunal and fecal gut microbiome responses [22]; and the butyrate supplementation in mitigating the LPS-induced changes in intestinal morphology, microbiome, energy utilization, and inflammation in pigs [39] were all likely mediated through gut endogenous AP functional plasticity; however, these interplay and connections were not established in these studies. Future research is needed to deepen our understanding of how dietary, therapeutic, and physiological factors regulate the host gut microbiome, digestive function, and whole-body physiology through regulating the endogenous AP functionality in pigs.
